# Fine mapping of complex traits in non-model species: using next generation sequencing and advanced intercross lines in Japanese quail

**DOI:** 10.1186/1471-2164-13-551

**Published:** 2012-10-15

**Authors:** Laure Frésard, Sophie Leroux, Patrice Dehais, Bertrand Servin, Hélène Gilbert, Olivier Bouchez, Christophe Klopp, Cédric Cabau, Florence Vignoles, Katia Feve, Amélie Ricros, David Gourichon, Christian Diot, Sabine Richard, Christine Leterrier, Catherine Beaumont, Alain Vignal, Francis Minvielle, Frédérique Pitel

**Affiliations:** 1 INRA, UMR444 Laboratoire de Génétique Cellulaire, Castanet-Tolosan, F-31326, France; 2 ENVT, UMR444 Laboratoire de Génétique Cellulaire, Toulouse, F-31076, France; 3 INRA, GeT-PlaGe Genotoul, Castanet-Tolosan, F-31326, France; 4 INRA, Sigenae UR875 Biométrie et Intelligence Artificielle, Castanet-Tolosan, F-31326, France; 5 INRA, Sigenae, Nouzilly, F-37380, France; 6 INRA, PEAT Pôle d'Expérimentation Avicole de Tours, Nouzilly, F- 37380, France; 7 INRA, UMR1348 PEGASE, Saint-Gilles, F-35590, France; 8 Agrocampus Ouest, UMR1348 PEGASE, Rennes, F-35000, France; 9 INRA, UMR85 Physiologie de la Reproduction et des Comportements, Nouzilly, F-37380, France; 10 CNRS, UMR6175 Physiologie de la Reproduction et des Comportements, Nouzilly, F-37380, France; 11 Université François Rabelais Tours, UMR Physiologie de la Reproduction et des Comportements, Nouzilly, F-37380, France; 12 INRA, UR83 Recherche Avicoles, Nouzilly, F- 37380, France; 13 INRA, UMR1313 Génétique animale et biologie intégrative, Jouy en Josas, F-78350, France; 14 AgroParisTech, UMR Génétique animale et biologie intégrative, Jouy en Josas, F-78350, France

**Keywords:** Quail, Tonic immobility, Sequencing, AFLP, Transcripts, SNP, AIL

## Abstract

**Background:**

As for other non-model species, genetic analyses in quail will benefit greatly from a higher marker density, now attainable thanks to the evolution of sequencing and genotyping technologies. Our objective was to obtain the first genome wide panel of Japanese quail SNP (Single Nucleotide Polymorphism) and to use it for the fine mapping of a QTL for a fear-related behaviour, namely tonic immobility, previously localized on *Coturnix japonica* chromosome 1. To this aim, two reduced representations of the genome were analysed through high-throughput 454 sequencing: AFLP (Amplified Fragment Length Polymorphism) fragments as representatives of genomic DNA, and EST (Expressed Sequence Tag) as representatives of the transcriptome.

**Results:**

The sequencing runs produced 399,189 and 1,106,762 sequence reads from cDNA and genomic fragments, respectively. They covered over 434 Mb of sequence in total and allowed us to detect 17,433 putative SNP. Among them, 384 were used to genotype two Advanced Intercross Lines (AIL) obtained from three quail lines differing for duration of tonic immobility. Despite the absence of genotyping for founder individuals in the analysis, the previously identified candidate region on chromosome 1 was refined and led to the identification of a candidate gene.

**Conclusions:**

These data confirm the efficiency of transcript and AFLP-sequencing for SNP discovery in a non-model species, and its application to the fine mapping of a complex trait. Our results reveal a significant association of duration of tonic immobility with a genomic region comprising the *DMD* (dystrophin) gene. Further characterization of this candidate gene is needed to decipher its putative role in tonic immobility in *Coturnix*.

## Background

The two avian *Coturnix* species - Japanese quail (*Coturnix japonica*) and Common quail (*Coturnix coturnix*) - belong to the order Galliformes and to the family *Phasianidae*, which also includes the chicken (*Gallus gallus domesticus*). The distributions of the two quail species overlap in the lake Baikal region, to and from where wild quail migrate every year, toward Asia for *C*. *japonica* and toward Europe and Africa for *C*. *coturnix*[1]. However, hybridization is possible between the two species [2], and hybrid males have a distinct crow [3].

In Asia, the Japanese quail was domesticated for its singing abilities in the 15th century, and selected lines were developed in Japan for egg production at the beginning of the 20th century [4]. Japanese quail lines were then introduced successively in North America, Europe and the Near and Middle East in the first half of the 20th century [5]. Its small size, short generation interval, good laying abilities and ease of reproduction interested scientists looking for a bird easy to maintain and to experiment with in the laboratory, or willing to develop alternative ways of producing eggs and meat for human consumption [5]. However, for unknown reasons, the Common quail was not domesticated. Research on Japanese quail has led to the development and use of commercial quail strains for meat and egg production in Asia, Europe and other parts of the world [6] and to a better knowledge of its genetics [7].

The first modern genetic tools developed specifically for quail analyses were panels of markers [8,9] and genetic maps using AFLP (Amplified Fragment Length Polymorphism) [10] and microsatellites [11]. Despite their limited extent, they have already been used extensively. At the population level, genome scans were carried out to detect QTL (Quantitive Trait Loci) for traits associated to fearfulness [12], growth, egg production and duration of tonic immobility [13] and egg laying curve [14], and markers were used to discriminate the two species and their hybrids [15,16]. At the genome level, genes carrying causal mutations were found for plumage colours, like *TYRP1* for the roux plumage [17] and *ASIP* for the yellow [18] and recessive black [19] plumages. More recently, the existing quail maps were integrated and aligned to the assembled chicken sequence [20], thereby confirming the high degree of synteny conservation expected because of the high level of chromosome homology observed between both species [21], and the relevance of quail models for trait analyses in poultry.

Further developments in quail genomics are held back by the small number of available markers which delay the search for other causal mutations. It is especially critical for traits with no known homologous phenotypes in chicken, like the rusty plumage colour and the curly-shaped feathers [22], and for the fine mapping of QTL. Our objective was to develop a dedicated quail SNP (Single Nucleotide Polymorphism) panel, in order to fine map a QTL for duration of tonic immobility. Tonic immobility (TI) is an innate fear-related behaviour which occurs throughout the Animal Kingdom [23,24] and has been studied extensively in poultry (see [25]). Its duration (dTI) is considered to be a measure of fearfulness, representing an antipredatory behaviour. In birds, it is characterized by a strong motor inhibition, elicited by a brief period of physical restraint. The animal first struggles and attempts to escape, then gets immobile even after termination of restraint, and this freezing-like posture may last from a few seconds to several hours [24]. Duration of tonic immobility is a quantitative polygenic trait with low to moderate heritability in poultry [26]. It has been successfully selected upwards and downwards in experimental quail lines over a large number of generations [27]. Despite its adaptive nature, fear may have deleterious effects on domestic animal welfare and health [25], and the relationship between duration of tonic immobility and productivity has been shown to vary from a positive to a negative one depending on the avian population and on the performance traits [28-30].

Deciphering the genetic architecture of tonic immobility will be challenging, as for other complex traits [31]. We first aim to fine map a QTL associated with dTI previously mapped to quail chromosome 1 by using two F2 crosses between a Japanese quail line selected for its long tonic immobility (LTI) and two lines with shorter tonic immobility, STI (Short Tonic Immobility, selected for lower tonic immobility [12,13]) and DD (selected for early egg production [12,13]). To this end, two Advanced Intercross Lines (AIL) were built. Provided the density of information is sufficient, using advanced generations is expected to restrict the genome region involved in the variability of the trait [32], with the ultimate objective to identify a causative gene. We chose to obtain a high number of SNP directly from our quail populations by high-throughput sequencing (Titanium 454 GS-FLX, Roche) of two main types of reduced representations of the genome: restriction digested fractions of genomic DNA, and EST (Expressed Sequence Tag), extending a first experiment [33] by increasing the number of sequences obtained and detecting reliable SNP. A subset of these markers was selected to analyse the AIL populations and to fine map the QTL.

## Results and discussion

### Sequence analyses and SNP discovery

The sequences obtained in this study are available at NCBI (http://www.ncbi.nlm.nih.gov/, accession number SRP002189).

Despite the rapid reduction of sequencing costs, identifying SNP by whole-genome sequencing of many individuals in a non-model species remains expensive, notably because the reliability of the identified SNP depends on the sequence depth [34]. Therefore, another approach was chosen [35] and reduced representation libraries (RRL) were built: a genomic library with tagged samples (AFLP fragments) and a transcriptomic library (cDNA fragments).

**Figure 1:**
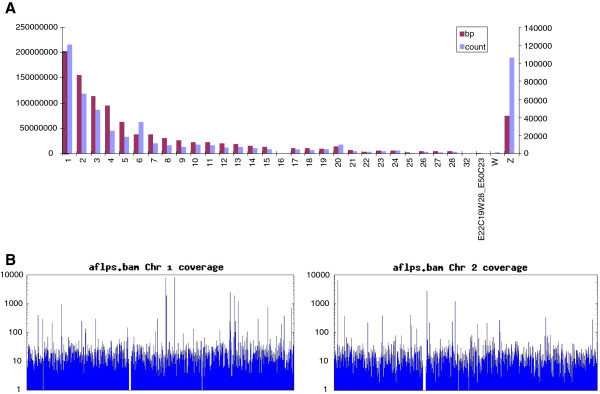
**Chicken genome coverage by quail AFLP fragments.A**. Number of reads aligned to each chicken chromosome, after Burrows-Wheeler Alignment algorithm (bwa bwasw analysis). For each chicken chromosome (X axis), chromosome length in base pairs (bp, Chicken May 2006 (WUGSC 2.1/galGal3) assembly) and number of mapped reads (count) is indicated (Y axis). **B**. Examples of chicken chromosome coverage by the quail reads for GGA01 (left) and GGA02 (right). X axis: position along the chromosome (physical length). Y axis: Number of reads aligned along the chromosome (counts, log scale).

AFLP fragments were sequenced in one and a half run which produced 1,106,762 reads, totalizing about 280 Mb of sequence. Reads were mapped to the chicken genome (Figure 1) to determine if the chicken genome coverage was compatible with our aim to select quail markers evenly distributed across the genome, through the use of the chicken sequence assembly. The average number of reads per chromosome is consistent with the chicken chromosome lengths, with the exception of the Z chromosome, where more reads are observed than expected. This is probably due to the repetitive nature of a large part of the sequence of the Z chromosome [36]. In total, 72% of the sequences mapped to the chicken genome. This result is comparable with that obtained in turkey, using a different alignment algorithm [37], confirming the possibility to obtain satisfactory results from alignment of heterologous sequences on a reference genome assembly. This illustrates the fact that both species have a similar level of divergence (around 35 MYA) with the chicken [20,38]. Except for some regions with high depth due to repetitive elements, the AFLP coverage was uniform enough (Figure 1b) to develop genome-wide SNP assays with confidence, given the proximity of the chicken and quail genomes [20].

From these data, 847,452 reads (76.6%) with identity tag and length higher than 120 bases were assembled into 42,860 contigs (N50 = 331 bp, consistent with the fraction of the AFLP fragments sequenced: between 200 and 400 bp), leaving 95,812 singletons. In the absence of a reference quail genome sequence, the SNP detection was performed from the obtained contigs. This led to the identification of 10,545 SNP.

Regarding the SNP type, a transition/transversion rate of 2.2/1 was observed. This value is similar to those reported for chicken (2.2/1), duck (2.3/1), and slightly higher than that for the great tit (1.7/1) [39,40]. Overall, our results confirm that reduced representation libraries from AFLP-derived fragments are successful tools for SNP detection. They also have been effectively used for phylogeographic studies [41-46].

In addition to the AFLP library, a second reduced representation of the genome was obtained from brain and embryo transcripts. The brain transcriptome may reflect a higher part of the genome than other tissues [47], and brain is probably the location of differential expression of behavioural genes between our quail lines. The embryo transcriptome was added to find SNP in genomic regions not covered by the brain transcriptome.

As previously described [33], a total of 399,189 cDNA reads have been obtained, of which 374,939 filtered sequences were assembled into 31,010 EST contigs, with an average length of 733 bases (from 100 to 6393, N50 = 786) and an average depth of 11 (from 2 to 3049), leaving 36,572 singletons. Among these 31,010 contigs, 27,919 (90%) mapped to the chicken genome.

Out of the 67,582 contigs or singletons built from cDNA data, 54% had significant matches (evalue < 10^-5^) on Swissprot, RefSeq_RNA or RefSeq_Protein databases. Sequences with more than 50% coverage with an identity level higher than 70% (n=18,416) corresponded to 8,550 unique referenced genes.

As already established for several species including *Brassica rapa*, *Drosophila melanogaster* or *Homo sapiens*[48-50], the transition/transversion ratio is higher in coding sequences than in untranscribed regions, and the 3.1:1 ratio in our EST data versus 2.2:1 from AFLP data confirmed this assessment. SNP were mainly discovered in non-coding region (Additional file 1: Figure S1), and 30% of the observed SNP in the coding region were non-synonymous SNP, like in chicken [51].

A total of 17,433 SNP were detected: 10,545 came from the genomic fragments while 6,888 were identified from the cDNA sequences, 29 of which being common between the two analyses based on their mapping on the chicken genome. SNP with at least 25 bp of flanking sequence each side have been submitted to dbSNP. This panel constitutes the first genome-wide collection of SNP markers for genetic studies in *Coturnix*.

### Tonic Immobility of AIL

The distribution of dTI was similar for the two AIL populations (Additional file 2: Figure S2). Yet, the distribution for DD-LTI was shifted slightly toward higher values of dTI, and was therefore more affected by the censoring of dTI at 300 sec. This difference probably reflects the distinct origin and history of selection of the two lines with lower dTI, DD (not selected on dTI) and STI (selected for short dTI) which were used to start the two AIL populations in crosses with Line LTI (selected for long dTI).

### QTL analyses

As previous experiments had shown the presence of a suggestive QTL on quail chromosome 1 [12,13], SNP were selected from sequences mapping to the homologous chicken chromosome 1. A total of 1,098 SNP selected from AFLP and 1,567 observed in cDNA were obtained, and 384 SNP putatively covering the entire quail chromosome 1 were chosen for genotyping. Twenty-five of them, detected from quail cDNA putatively homologous to several chicken genes, appeared to map on different chromosomes when considering their best localization (Additional file 3: Table S1). Out of the 384 markers, 352 (92%) gave reliable genotypes in our populations (AIL_DD: 326 SNP, AIL_STI: 336 SNP), and a total of 320 (91%) were polymorphic. After testing for non deviation from Hardy-Weinberg equilibrium, respectively 305 (AIL_DD) and 332 (AIL_STI) markers were used for further analyses.

**Figure 2:**
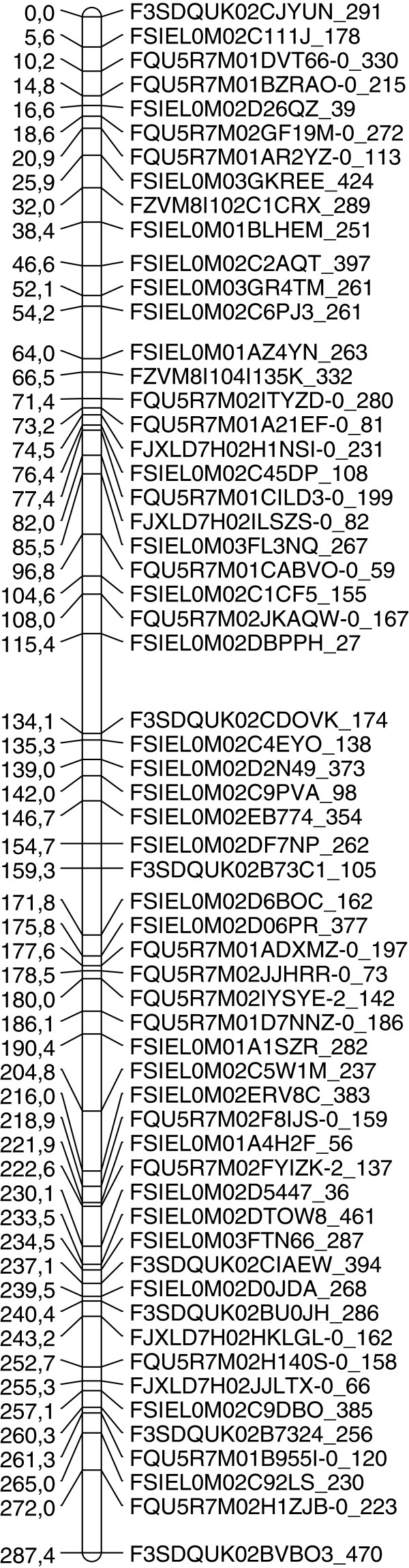
**Genetic framework map of quail chromosome 1.** Positions are given in cM.

A framework map of quail chromosome 1 was built, comprising 60 markers with an average number of informative meioses of 505 (from 79 to 835), for a genetic length of 287.4 cM and with an average between-marker distance of 4.9 ± 3.9 cM (Figure 2). The length of our genetic map is comparable to that (274.8 cM) obtained previously for the same chromosome by Kayang and coworkers [20], but is shorter than the one (402.9 cM) obtained in a backcross study [52]. Several factors could explain this difference in length, like the structure of the populations and the number of individuals genotyped in each study, the type of markers used or the method employed to calculate the distance.

Assuming quail and chicken chromosomes 1 have similar physical length, the relative coverage for our quail genetic map was 1.5 cM/Mb, which is much lower than the 2.4 cM/Mb ratio observed in chicken [53]. This result, probably partially related to the variability of recombination rates between populations, is consistent with the smaller number of chiasmata reported in quail lampbrush chromosomes [54].

AIL populations are expected to offer a very powerful tool to fine map QTL, especially when inbred lines are used as founders [32], and the final AIL generation is analyzed using classical QTL methods, taking advantage of the recombination events that have occurred over the course of multiple successive generations. We used this strategy with our lines to fine map a QTL previously detected in two distinct F2 crosses [12,13]. Two AIL populations were used: the AIL_DD families based upon F2 crosses of DD and LTI quail, and the AIL_STI population resulting from F2 crosses between STI and LTI birds.

**Figure 3:**
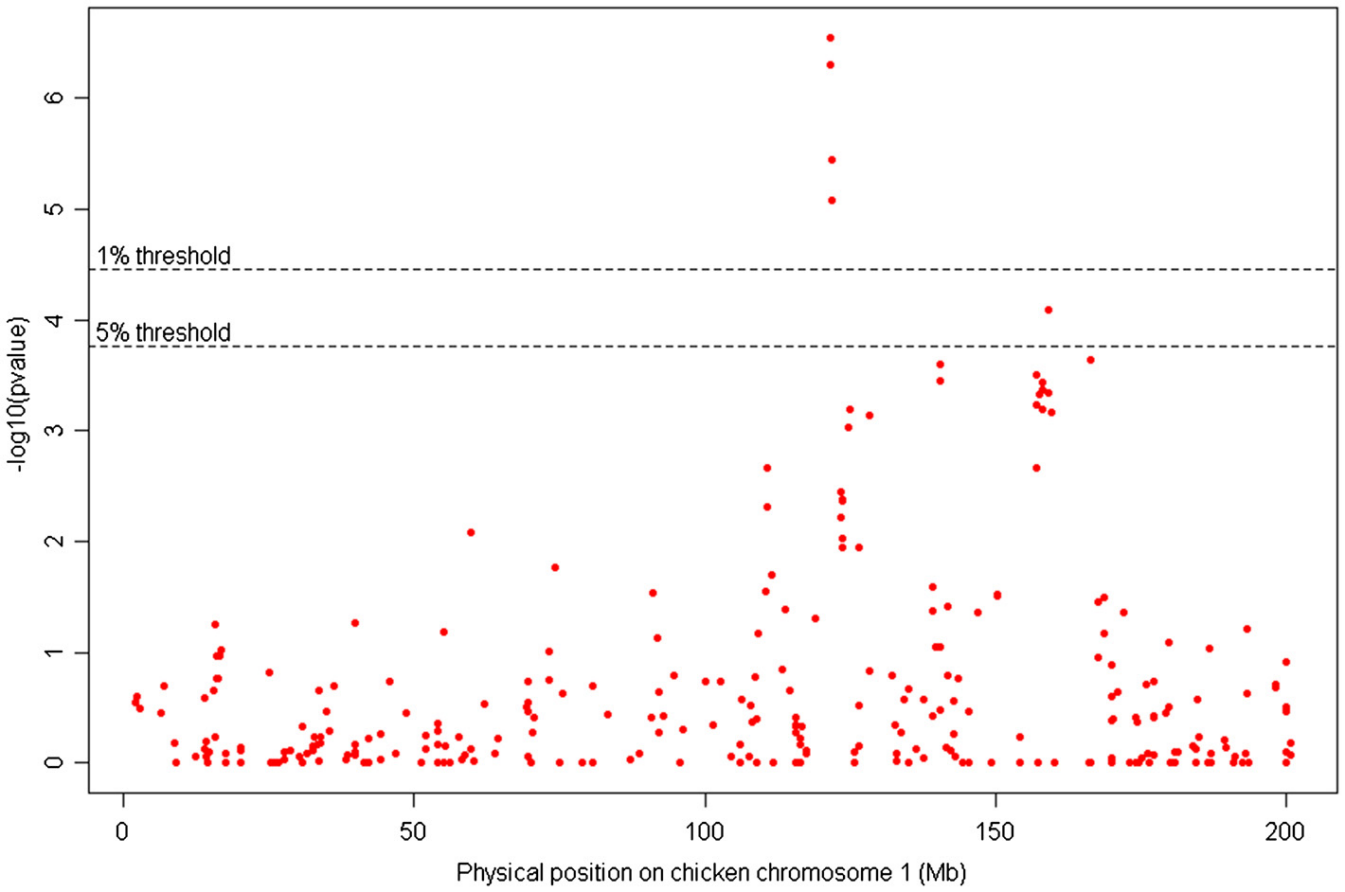
**-log10**(**p**_**value**)** plot of the association study of tonic immobility in the AIL**_**DD population.** Each SNP is mapped to its orthologous position on chicken chromosome 1.

First, QTL detection was applied within AIL sire families by interval mapping with QTLMap [55]. Unexpectedly, no QTL for dTI could be detected in either of the final AIL generations, even after performing a bulk analysis using the two AIL populations. We suspected this result was the consequence of a lower detection power because the origin of alleles was not accounted for. Indeed, recent AIL studies included genotyping and phenotyping of all individuals in the AIL line [56], or were set up from at least one inbred line [57], which made it possible to trace the origin of alleles back to the F0 lines. To overcome the problem, an association analysis was performed in our AIL, with the assumption that enough power would be gained by adding information from dams within families of sires homozygous for the QTL, that were uninformative in the QTL mapping analysis based on familial linkage. This strategy was successful in the AIL_DD population (Figure 3): 4 markers, in a genomic region orthologous to position 121.54-121.78 Mb in chicken, showed a significant association with dTI (Bonferroni corrected for multiple testing p < 0.01): FSIEL0M02C8AXD_286, FSIEL0M02D00IT_258, FSIEL0M02C7RBX_180, and FSIEL0M02DMFKD_281 (Additional file 3: Table S1). Non-significant markers encompassing the region were respectively at 119.07 (F3SDQUK02B73C1_105) and 123.28 Mb (FSIEL0M01A1W48_163). As the AIL approach allows us to reasonably consider that linkage disequilibrium is limited, our investigation could be first restricted around this 4.21 Mb area. This result confirms the first mapping of a suggestive QTL in the original F2 population, as the region detected here belongs to the confidence interval estimated by the two-LOD drop-off method [58] from the original LTI-DD QTL position [12,13,58]. The same region also contained a significant QTL in the original LTI-STI F2 population [12], but had not been previously shown to be associated with dTI in chicken [59].

The association study showed also that the most significant marker (FSIEL0M02C8AXD_286, orthologous position 121.54 Mb on the chicken genome) had a substitution effect of 0.45 phenotypic standard deviations and that only 3 F6 sires were heterozygous at the QTL. By using these estimates, the power of detection of the QTL in the AIL_DD familial analysis was estimated to reach only 40%, which accounts for the fact that the QTL was not detected using this approach.

The same analysis in the AIL_STI population did not reveal any significant association between dTI and a marker. This may be due to the lack of linkage disequilibrium between the markers and the causative mutation in the STI population: whereas the most significant markers from the AIL_DD were fixed in the DD line (all F0 parents homozygous for the same allele), the STI population showed allele sharing, indicating that a complete association between a putative fixed mutation and a SNP marker could not be observed in the AIL_STI. The facts that the AIL were not phenotyped under the same setting (housing, technician) and that the genetic origins of the F0 were a same base line population for LTI and STI, and a different unrelated one for DD [60] may have also contributed to the diverging results of the association analyses.

Most of the genes recorded in the investigated region have no known association with tonic immobility (Additional file 4: Table S2), with the notable exception of the Dystrophin gene (Q9PTS7_CHICK or *DMD*). In the candidate region, one gene (*IL1RAPL1*, IL-1-receptor accessory protein-like 1, 119.54-120.16 Mb) is involved in mental retardation or autism [61,62]; structural variations including another gene (*PTCHD1*, patched domain containing 1, 121.89-121.94 Mb) have been shown to be involved in mental disorders like autism [63], but no clear connection could be made with tonic immobility. The 4 significant markers are included in 4 different genes (Additional file 4: Table S2): *Q5ZLT2*, or *PDK3* (pyruvate dehydrogenase kinase, isoenzyme 3), a kinase involved in the regulation of glucose metabolism, has been showed to be associated with drug resistance and colon cancer [64]; *EIF2S3* (eukaryotic translation initiation factor 2, subunit 3 gamma) is a gene coding for a subunit of eukaryotic translation initiation factor 2, involved in protein synthesis by playing a role in translation start site selection (see [65]); *KLHL15* (kelch-like protein 15) encodes for a protein belonging to the Kelch-like proteins family, implicated in embryogenesis and carcinogenesis mainly through cytoskeleton organization [66]; all these three genes do not seem to have a function directly linked with the duration of tonic immobility. The fourth one, *SAT1* (spermidine/spermine N1-acetyltransferase 1), catalyzing the acetylation of polyamines, has been associated with antineoplastic activity of several antitumor [67] and is essential for the polyamine homeostasis (see [68]). *SAT1* has been shown in transgenic mice to be involved in several behavioural traits as activity and spatial learning in females, but not in defensive reactivity [69]. It may constitute a correct candidate gene. But the *DMD* gene, localized between 118.07 and 119.07 Mb, seems to be a very interesting one even if only the 3’end of the gene is included in the candidate region. Sekiguchi *et al*. [70] showed that *DMD* is involved in defensive behaviors in mouse, including a strong freezing response, reminiscent of the strong motor inhibition exhibited by quail in the tonic immobility response. This gene, well known for its implication in Duchenne muscular dystrophy in Human through mutations leading to loss-of-function of the gene products in striated muscles [71], is also expressed in the brain, where it might account for the mental deficiency reported in Duchenne muscular dystrophy patients (see [72] for a review). However, further work is needed to investigate the precise involvement of brain dystrophin in the control of emotions and cognition.

## Conclusions

Using Japanese quail as an example, this work confirms the efficiency of AFLP fragments as a reduced representation of the genome to develop SNP markers for species for which no whole-genome draft sequence is available. Transcript sequencing brings a high number of quail EST that can be used to help annotate a future quail genome assembly. This study provides also a high number of Japanese quail SNP, for genetic analyses in *Coturnix*. It underlines the importance of modern technologies as Next Generation Sequencing in the renewed ability to perform association analyses, even in non-model species.

The association study carried out with a SNP subpanel for chromosome 1 led to a candidate gene, *DMD*, as being partly responsible for the variability of tonic immobility in Japanese quail. It should be the focus of further investigations to help unravel this complex trait.

## Methods

### Experimental animals

Tonic immobility was the selection criterion for the divergent selection of two quail lines: the LTI and STI lines [27]. These two lines and a third one, line DD unselected on tonic immobility and of a different origin [73], were used for the study. Two AIL populations were produced: the "AIL_DD" population was obtained by crossing 6 DD male to 6 LTI females and 6 LTI males to 6 DD females to produce the F1 generation. The offspring were then intermated within each generation until the F7 generation (603 offspring, Table 1). The "AIL_STI" population was founded by crossing LTI and STI quails and carried out until the F6 generation (679 offspring, Table 1). Between 9 and 11 days of age, duration of tonic immobility was measured on each quail of the final AIL generation. TI duration is the time during which a bird remains immobile after the fear reaction has been induced by keeping it on its back in a U-shaped cradle for 10 seconds (Additional file 5: Figure S3). dTI was set to 0 second if tonic immobility was not induced after five attempts. If the quail was still in a tonic immobility state after 5 min, the observation was censored and dTI set to 300 seconds. Statistical analyses were run on the transformed variable LOG(dTI+1).

**Table 1:** Number of animals in each generation of the AIL pedigrees

**Generation**	**AIL**_**DD**			**AIL**_**STI**		
	**Offspring**	**Sires**	**Dams**	**Offspring**	**Sires**	**Dams**
**F0**	24	/	/	72	/	/
**F1**	40	12	12	72	36	36
**F2**	433	10	26	72	36	36
**F3**	96	14	38	72	35	35
**F4**	98	48	48	72	36	36
**F5**	98	48	48	71	32	32
**F6**	52	35	35	679	10	30
**F7**	603	13	39	/	/	/

### 454 sequencing

Transcripts and AFLP fragments libraries' preparation, and 454 sequencing, have already been described [33]. Three quail lines were used: LTI, STI, DD [27,73], and total RNA was extracted from about 500 mg of adult quail brains (4 samples from each line) and total embryos (Embryonic day 8, 2 samples from each line) for library preparation. Blood samples from two individuals from each line were used for the AFLP library preparation.

### Sequence analyses

Trimming of low-quality sequence and 454 adaptors were performed using the Newbler software (Roche/454).

#### AFLP analyses

Identification of tags within each sequence was performed by using crossmatch (from the PhredPhrap suite, http://www.phrap.org/phredphrapconsed.html, Phil Green) with tagged AFLP primers as query sequences. The crossmatch results were used to split the initial sequences when primers were observed within the fragment sequence (and not only at its ends). We then selected the sequences long enough for the clustering step (>120 bp). Sequences with 1 or 2 tags were used to generate sequence clusters, based on more than 95% identity (over a length of 80% of the shorter sequence) between cluster fragments, as processed by Megablast [74]. Next, contigs were assembled from the clusters with the CAP3 sequence assembly program [75].

SNP detection was performed with an in-house script: we selected SNP with a minimum depth of 5 sequences, a minus allele count of 2 and having complete similarity between the sequences in the ten base pairs located on the left and right sides of the SNP.

Alignment of the reads to the chicken genomic sequence (Chicken May 2006 (WUGSC 2.1/galGal3) assembly) was performed with the Burrows-Wheeler Alignment algorithm bwa, version 0.6.1-r104, command bwasw, at default parameters (mismatch penalty of 3, maximum one gap opened, gap open penalty of 11, gap extension penalty of 4…). This algorithm, more permissive about alignment gaps, has been developed for long queries but was used here to deal with heterologous alignment [76]. Genome coverage was calculated from the SAMtools v0.1.18 (mpileup tool) utilities [77] (samtools mpileup -A -B) and visualised through a custom script.

#### cDNA analyses

Artefactual sequence duplicates due to the 454 technology (98% identity, same start, same strand, same 454 run) were removed [78].

Because of the large number of sequences, we used a two-step process to assemble these sequences into contigs. The first step built clusters of sequences sharing at least 75 bp with an identity rate of 96% using MegaBlast [74]. The second step constructed coherent contigs from the previous clusters using CAP3 [75], at the recommended stringency of 40 bp overlap with 90% sequence identity.

As no annotated quail reference genome is available, the contigs or singletons were annotated by BLAST [79] on the chicken genome using several databases (SwissProt, Refseq_RNA, RefSeq_Prot). Sequences with more than 50% coverage and with an identity level higher than 70% were considered as homologous to a reference gene, and their GO annotation were retrieved from the databases. All data were loaded in a locally-adapted Ensembl database.

The SNP detection was performed with the same script and parameters as for the AFLP fragments. Functional annotation of the SNP was performed with custom Perl scripts, using the Ensembl APIs (Application Programme Interface) [80].

### Marker genotyping

In order to select SNP from contigs uniquely mapped to GGA01, a Megablast analysis [74] was performed to map the AFLP contigs on the chicken reference genome (Chicken May 2006 (WUGSC 2.1/galGal3) assembly). Similarly, cDNA SNP were selected from genes localized on chicken chromosome 1. Then, by using their orthologous positions on the chicken genome, 384 SNP were chosen as putatively covering the entire quail chromosome 1 (Additional file 3: Table S1). They were analysed on 1399 animals (AIL F6 or F7, their parents, and 26 F0 individuals, corresponding to the 24 AIL_DD founders and 2 LTI individuals) using the Illumina GoldenGate assay. Genotype calling was performed with GenomeStudio V2010.1 (Illumina). Genotypes were filtered for call rate (> 0.85), call frequency (> 0.50) and cluster dispersion. Markers with an unexpected deviation from Hardy-Weinberg equilibrium (p < 10^-6^) were removed with PLINK (option -- hardy, http://pngu.mgh.harvard.edu/purcell/plink/ ) [81].

### QTL analyses

#### Interval mapping

A genetic map of quail chromosome 1 was built by using the CRI-MAP program [82] version 2.503 (revised version of Phil Green's CRI-MAP v2.4, modified by Jill Maddox and Ian Evans) with the AIL_STI population, which had a higher number of reliable markers. The "twopoint" option allowed to group markers in a unique linkage group (twopoint LOD score >= 3). The “build” option was used to order markers within the linkage group, while the “flips” option enabled to confirm the order of the markers. We build a framework map with a LOD threshold of 3, using a stepwise locus-adding strategy, starting from the markers with the higher number of informative meioses.

QTL interval mapping was performed with the QTLMap software using likelihood computation at every centimorgan, ignoring the maternal chromosomes [55]. The linearized likelihood under the alternative hypothesis was computed as:

$$\begin{array}{c} L_{x}=\Sigma_{i}\Sigma_{k}f\left[y_{\mathrm{ik}}/hs_{i},M_{i}\right],\\ \;\text{with}\;f\left[y_{\mathrm{ik}}/hs_{i},M_{i}\right]∼N\left(\Sigma_{p}b_{p}\alpha_{p}+\mu_{i}+P_{\mathrm{ik}}^{x}a_{i}^{x};\sigma_{i}\right)\text{,} \end{array}$$

with *i* the sire family in the design; *ik* the progeny within sire family *i*; *fy*_*ik*_/*hs*_*i*_, *M*_*i*_ the likelihood of individual *ik* phenotype computed conditionnally to *hs*_*i*_ the sire *i* phase and *M*_*i*_ the marker information in the sire family *i*, considered as normally distributed with mean comprising some nuisance fixed effects *α*_*p*_, the sire family mean of the phenotypes *µ*_*i*_, and the QTL substitution effect in sire family *i a*_*i*_^*x*^, and standard deviation of the sire family *i σ*_*i*_ (heteroscedastic model). *P*_*ik*_^*x*^ is the transmission probability of one of the sire chromosome to its progeny *ik* at position *x*. The technician having recorded the phenotypes, the sex and the hatch were set as nuisance fixed effects in the model. Chromosome-wide significance thresholds at 5% were calculated through 1,000 phenotype permutations. Power of detection was tested on AIL_DD modulating its two principal components, QTL frequency (ie proportion of heterozygote sires in the parental F5 generation) and QTL effect, by simulating QTL using QTLMap. 

#### Association analyses

Marker-trait association was tested by using a linear mixed model:

$$y=X\beta +Z_{1}a+Z_{2}b+e$$

where

*β* : fixed effects (intercept, hatch, sex, technician, genotype at marker)

*a* : random sire effect on trait mean a~N(0,**I**σ^2^_a_)

*b* : random dam effect within sire on trait mean b~N(0,**I**σ^2^_b_)

*e* : residual e~N(0,**I**σ^2^_e_)

where ***X****,****Z***_***1***_ and ***Z***_***2***_ are design matrices and ***I*** denotes the identity matrix.

The model was implemented using the R « nlme » package (*Linear and Nonlinear Mixed Effects Models*) [83]. The Bonferroni correction was applied to correct for multiple testing.

#### Candidate gene identification

A search for candidate genes was performed through AnnotQTL [84] (http://annotqtl.genouest.org). This software gathers the functional annotation of genes mapped to a specific chromosomal region, from several public databases (Gene Ontology, Mammalian Phenotype, HGNC and Pubmed), in order to help find the best candidate genes.

## Competing interests

The authors declare that they have no competing interests.

## Authors’ contributions

CB, AV, FM and FP conceived the study. FM, CD and FP participated in its design and coordination. CB, FM, SR and DG provided the AIL pedigrees. CL, FM, SR and CB supervised phenotyping. SL, KF, FV, OB and LF carried out the molecular studies and analysis. PD, CK, CC and AR performed the bioinformatic studies. SL and LF conducted SNP analyses. LF, BS and HG performed QTL and association studies. LF, FM and FP drafted the manuscript. All authors read and approved the final manuscript.

## Supplementary Material

Additional file 1 **Figure S1.** Distribution of the functional annotation of the detected SNP (log scale). Annotations are from the Ensembl APIs (Application Programme Interface): the functional consequence of each SNP in each transcript has been predicted using the Variant Effect Predictor (VEP). All the non intergenic consequences are represented. Click here for file

Additional file 2 **Figure S2.** Distribution of the trait in both AIL populations. TI : Tonic Immobility. Click here for file

Additional file 3 **Table S1.** List of the 384 SNP used in this study. Click here for file

Additional file 4 **Table S2.** List of the genes localized in the candidate interval. Source : Biomart, Ensembl Genes 61 (Sanger UK), Gallus Gallus Genes (WASHUCS52). Click here for file

Additional file 5 **Figure S3.** Quail in a tonic immobility state. Immobilization is induced by keeping the animal on its back in a U-shaped cradle for 10 seconds. Click here for file
