# Supplementary material for: Fine mapping of complex traits in non-model species: using next generation sequencing and advanced intercross lines in Japanese quail
Source: BMC Genomics. 2012 Oct 15;13:551. doi: 10.1186/1471-2164-13-551 (PMC3534603; doi:10.1186/1471-2164-13-551)
Supplement: Additional file 1 — Figure S1. Distribution of the functional annotation of the detected SNP (log scale). Annotations are from the Ensembl APIs (Application Programme Interface): the functional consequence of each SNP in each transcript has been predicted using the Variant Effect Predictor (VEP). All the non intergenic consequences are represented. [file 1471-2164-13-551-S1.pptx]

## Slide 1
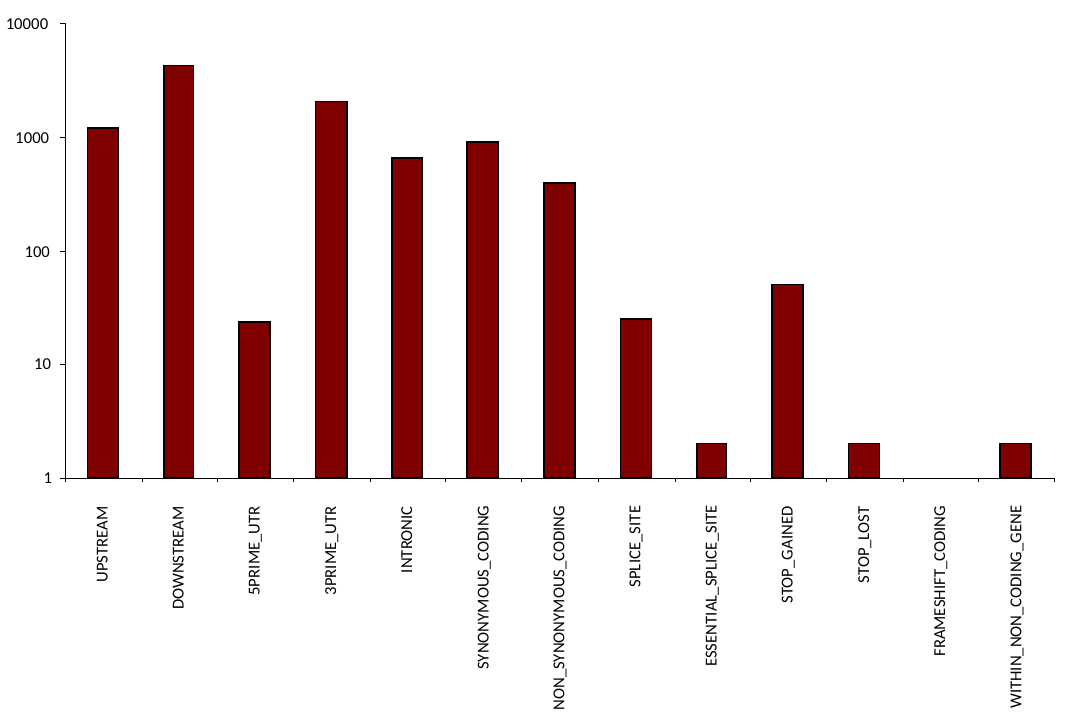

10000
1000
100
10
1
INTRONIC
UPSTREAM
STOP_LOST
SPLICE_SITE
5PRIME_UTR
3PRIME_UTR
STOP_GAINED
DOWNSTREAM
FRAMESHIFT_CODING
ESSENTIAL_SPLICE_SITE
SYNONYMOUS_CODING
WITHIN_NON_CODING_GENE
NON_SYNONYMOUS_CODING
