# Supplementary figures and images for: Fine mapping of complex traits in non-model species: using next generation sequencing and advanced intercross lines in Japanese quail
Source: BMC Genomics. 2012 Oct 15;13:551. doi: 10.1186/1471-2164-13-551 (PMC3534603; doi:10.1186/1471-2164-13-551)

## Slide 1
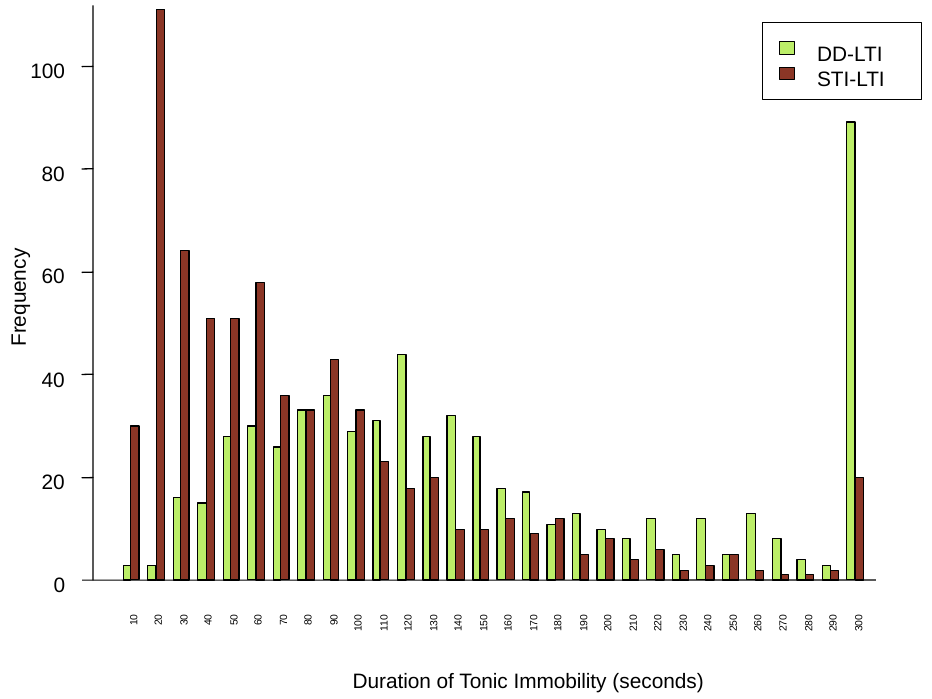

DD-LTI
100
STI-LTI
80
60
Frequency
40
20
0
10
20
30
40
50
60
70
80
90
100
110
120
130
140
150
160
170
180
190
200
210
220
230
240
250
260
270
280
290
300
Duration of Tonic Immobility (seconds)

Supplement: Additional file 2 — Figure S2. Distribution of the trait in both AIL populations. TI : Tonic Immobility. [file 1471-2164-13-551-S2.pptx]

## Slide 1
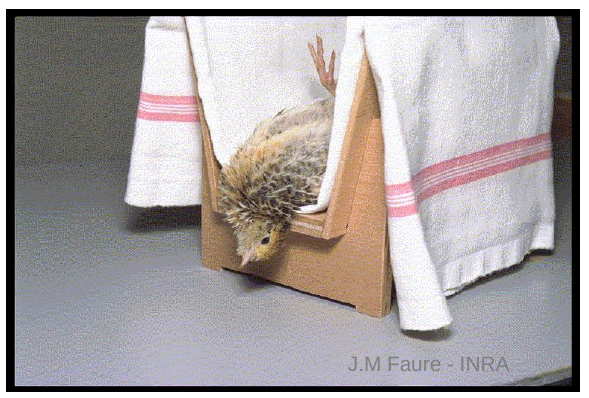

J.M Faure - INRA

Supplement: Additional file 5 — Figure S3. Quail in a tonic immobility state. Immobilization is induced by keeping the animal on its back in a U-shaped cradle for 10 seconds. [file 1471-2164-13-551-S5.pptx]
